# Tumour necrosis factor inhibitors reduce aortic stiffness progression in patients with long-standing rheumatoid arthritis

**DOI:** 10.1186/s13075-021-02546-3

**Published:** 2021-06-03

**Authors:** Alessandro Giollo, Giovanni Cioffi, Federica Ognibeni, Giovanni Orsolini, Andrea Dalbeni, Riccardo Bixio, Giovanni Adami, Angelo Fassio, Luca Idolazzi, Davide Gatti, Maurizio Rossini, Ombretta Viapiana

**Affiliations:** 1 Rheumatology Section, Department of Medicine, University of Verona Hospital Trust, Policlinico G.B. Rossi 10, 37134 Verona, Italy; 2 Division of Rheumatology, University of Padova, Padua, Italy; 3 Division of Cardiac Rehabilitation, S. Pancrazio Hospital, Arco di Trento, Trento, Italy; 4 Internal Medicine and Hypertension Section, Department of Medicine, University of Verona Hospital Trust, Verona, Italy

## Abstract

**Background:**

Aortic stiffness index (AoSI) has to be considered a proxy outcome measure in patients with rheumatoid arthritis (RA). The aim of this study was to comparatively describe AoSI progression in two groups of RA patients on long-term treatment with conventional synthetic disease-modifying anti-rheumatic drugs (csDMARDs) with or without tumour necrosis factor inhibitors (TNFi).

**Methods:**

AoSI was evaluated by Doppler echocardiography at the level of the aortic root, using a two-dimensional guided M-mode evaluation. Eligible participants were assessed at baseline and after 12 months. Changes in serum lipids, glucose and arterial blood pressure were assessed. All patients who did not change DMARD treatment during follow-up were consecutively selected for this study.

**Results:**

We included 107 (64 TNFi and 43 csDMARDs) RA patients. Most patients (74%) were in remission or low disease activity and had some CVD risk factors (45.8% hypertension, 59.8% dyslipidaemia, 45.3% smoking). The two groups did not differ significantly for baseline AoSI (5.95±3.73% vs 6.08±4.20%, *p*=0.867). Follow-up AoSI was significantly increased from baseline in the csDMARDs group (+1.00%; *p*<0.0001) but not in the TNFi group (+0.15%, *p*=0.477). Patients on TNFi had significantly lower follow-up AoSI from baseline than the csDMARDs group (−1.02%, *p*<0.001; ANCOVA corrected for baseline AoSI, age and systolic blood pressure). Furthermore, follow-up AoSI was significantly lower in TNFi than in csDMARDs users with an increasing number of CVD risk factors.

**Conclusion:**

Long-term treatment with TNFi was associated with reduced aortic stiffness progression in patients with established RA and several CVD risk factors.

## Introduction

Rheumatoid arthritis (RA) is a chronic immune-mediated and inflammatory disease characterized by a 48% increased risk of cardiovascular (CV) events and a 50% higher incidence of cardiovascular disease (CVD)-related mortality compared with the general population [1, 2].

There is growing evidence that increased arterial stiffness may account for the excess risk of CVD in RA [3–8]. Arterial stiffness is one of the earliest detectable manifestations within the atherosclerotic vessel wall [9, 10], and it acts as a strong independent predictor of CV events and all-cause mortality in various populations [11]. When structural and functional changes of the elastic fibres within the arterial wall occur, arteries progressively lose their low-stretch bearing component, longitudinal elasticity and geometry, leading to collagen deposition with decreased elasticity and stiffness, elongation and increased tortuosity [12]. While this phenomenon is strictly related to ageing, it can also be accelerated with increased CVD risk factors and inflammation (early vascular ageing). Arterial stiffness eventually results in higher driving pressures and increased energy demands for the heart, while leading to higher diastolic-systolic pressure differences (i.e. widening of pulse pressure). Increased arterial pressures and pulsatility impose higher mechanical stress on the vessels and organs, leading to strong associations between arterial stiffness and organ damage in the heart, kidney or brain [13].

With the aorta being the major elastic vessel in the body, aortic stiffness likely represents the most informative measurement of arterial stiffness [13]. Amongst the several principles, techniques and devices that have been proposed to measure arterial stiffness in humans, Doppler echocardiography is one of the cheapest, fast, widely available and reliable methods to assess aortic stiffness. Moreover, it can be easily integrated into a routine echocardiography assessment.

Aortic stiffness was significantly increased in RA patients [14–16], and it was associated with worse CVD outcomes [17]. Interestingly, treatment with conventional synthetic disease-modifying anti-rheumatic drugs (csDMARDs) or tumour necrosis factor inhibitors (TNFi) appeared to be the effective strategy to improve aortic stiffness in early RA patients [14, 18, 19].

Most clinical trials have been successful at demonstrating a beneficial effect of csDMARDs and TNFi on CV outcomes in RA of short duration [20–22], when CVD risk profile is still favourable and inflammation is at its highest. However, patients with RA are subject to great accumulation of CVD risk factors in a disproportionate manner than the general population and this can happen even when RA patients receive long-term therapy with good outcomes in terms of disease activity control [23–26]. In such patients with long-standing and established disease, whether csDMARDs and TNFi can still have an effect on aortic stiffness is largely unknown. This knowledge could encourage retention of csDMARDs or TNFi for their CV benefit beyond the control of inflammation. The aim of this study was to comparatively describe aortic stiffness progression in such RA patients treated with csDMARDs and TNFi.

## Methods

### Study protocol

This was a post hoc analysis of a wider cross-sectional study of cardiovascular assessment of non-institutionalized individuals >18 years of age affected with rheumatic and musculoskeletal diseases, which was started in 2014 at the Division of Rheumatology, Department of Medicine, University and Azienda Ospedaliera Universitaria Integrata of Verona (Italy). The study was approved by the institutional review board of the University of Verona (1707CESC) and conformed to the ethical guidelines of the Declaration of Helsinki as revised in 2000. All patients gave written informed consent signing a specific institutional consent form. Inclusion criteria comprised a diagnosis of RA according to the 2010 ACR/EULAR definition. Furthermore, we consulted medical notes to assess whether significant changes in medications had been recorded, so that all patients who did not change DMARD treatment during follow-up were selected for the study. The following exclusion criteria were applied: previous CVD diagnoses, events, and procedures (any known CVD including myocardial infarction, stroke, coronary revascularization, transient ischaemic attack, hospitalization for unstable angina, peripheral artery disease, symptomatic carotid artery disease, and uncontrolled systemic arterial hypertension). Patients who had started CV or RA medications within 6 weeks from the first assessment were also excluded from this analysis. All participants underwent an evaluation of cardiovascular risk factors and were offered echocardiography examination, carotid ultrasound and aortic stiffness assessment. Follow-up and instrumental assessments were performed yearly.

### Patient disposition

Participants were consecutively screened and recruited from March 2014 to March 2016. All recruited patients underwent a clinical evaluation by senior rheumatologists (OV, DG, LI) including assessments of disease activity, disease duration, body weight and height, medical history and CV and RA medications. Recruited patients were then referred for aortic stiffness assessment that was performed within 2 weeks. Laboratory tests including inflammatory markers, serology, lipids and glucose levels were performed within 2 weeks before or after aortic stiffness assessment. Follow-up assessments at 12 months were performed between March 2015 and March 2017. All patients in the TNFi group received a combination of csDMARDs and TNFi.

### Cardiovascular disease risk factors

The following CVD risk factors were collected: age; gender; systolic blood pressure (SBP), diastolic blood pressure (DBP) and heart rate were measured at the end of echocardiographic evaluation in supine position; weight and height with the calculation of body mass index (BMI); lipids including total cholesterol, low-density cholesterol and high-density cholesterol, and triglycerides; waist circumference; renal function; and smoking status. We defined obesity when body mass index (BMI) ≥ 30 kg/m^2^. Dyslipidaemia was defined as levels of total serum cholesterol >190 mg/dL and or triglycerides >150 mg/dL or pharmacologically treated high lipid serum levels. To assess renal function, we considered the glomerular filtration rate (GFR) estimated with the CKD-EPI equation and defined renal dysfunction as estimated GFR < 60 ml/min 1.73 m^2^.

### Rheumatoid arthritis-associated factors

Data on disease duration, anti-citrullinated peptides antibodies (ACPA) and rheumatoid factor (RF) were collected. Serum biomarkers of RA-related inflammation (C-reactive protein CRP, and ESR) were measured. RA disease activity was evaluated by the clinical disease activity index (CDAI) score and disease-activity score in 28-joints (DAS28). Patients were defined as having remission, low, moderate or high disease activity according to CDAI values. Current immunomodulating agents including conventional synthetic DMARDs and biologic DMARDs, glucocorticoid use and dose (in prednisone-equivalent milligrams daily), and NSAID use were recorded.

### Aortic stiffness assessment

Aortic stiffness was evaluated by Doppler echocardiography. All Doppler echocardiographic studies were performed by an expert sonographer (FO) using an Alpha Esaote Biomedica machine (Florence, Italy) equipped with a 2.5–3.5 MHz annular-array transducer and following a standardized protocol. Images were stored on compact disks or magneto-optical disks and forwarded for final interpretation to a senior cardiologist (GC) blinded to the identity of the subject. Aortic stiffness was assessed at the level of the aortic root, using a two-dimensional guided M-mode evaluation of systolic (AoS) and diastolic (AoD) aortic diameters, 3 cm above the aortic valve together with blood pressure measured by cuff sphygmomanometer. AoD was obtained at the peak of the R wave at the simultaneously recorded electrocardiogram, while AoS was measured at the maximal anterior motion of the aortic wall [27, 28]; for each diameter, five measurements were averaged. Values of SBP, DBP, AoS and AoD were used to calculate the aortic stiffness index (AoSI) using the following validated formula: AoSI = ln(SBP/DBP)/(AoS-AoD)/AoD. Intraclass correlation coefficient (ICC) with a two-way random model was used to assess the absolute reliability of aortic diameters and BP measurement in 50 patients. ICC values (95% CI) were 0.91 (0.86–0.94) for AoS, 0.93 for AoD, 0.92 for SBP and 0.94 for DBP respectively. ICC for calculated AoSI was 0.92.

### Statistical analysis

Continuous data are reported as mean values ± standard deviation (SD) or absolute numbers (percentage) for categorical variables. Treatment group comparisons of categorical variables were performed by chi-squared or Fisher test as appropriate; for continuous variables, independent samples T-test was used. Paired samples T-test was used to determine significant changes from baseline of continuous variables including AoSI, arterial blood pressure, lipids, glucose, inflammatory markers and RA disease activity scores. Treatment group comparisons of follow-up AoSI were performed in the whole study population using two-way analysis of covariance (ANCOVA) with Sidak’s correction for multiple comparisons, with treatment group and the number of CVD risk factors categorized into three groups (0–1, 2–3, or >3) as factors, and baseline AoSI, age and SBP as covariates. The choice of covariates was made upon prior data from our study [17]. All analyses were performed using the statistical package SPSS 22.0 (SPSS Inc. Chicago. Illinois), and statistical significance was identified by two-tailed *p* < 0.05. Figures were obtained using the GraphPad Prism software version 7.00.

## Results

### Baseline characteristics of csDMARDs and TNFi patients

The study population consisted of 107 white RA individuals, 43 patients in the csDMARDs group and 67 in the TNFi group. All patients had established RA and disease duration longer than 2 years. Most patients (74%) were in remission or low-disease activity, while disease activity was moderate only in 26% and high in none. High values of ESR (>40 mm/h) or CRP (>10 mg/L) were found in 11.8% and 8% only, respectively. Excluding age and sex, 92% of RA patients had at least one CVD risk factor, 58% two or more and 26% three or more. There were more than two CV risk factors in 28.6% of csDMARDs and 29.0% of TNFi groups, respectively (*p*=0.469). Patients in the csDMARDs and TNFi groups were equally balanced for the proportion of CVD risk factors and medications, and there were no significant differences in baseline SBP, DBP or serum lipids. The proportions of patients taking angiotensin-converting enzyme and angiotensin II receptor blockers, calcium channel antagonists, diuretics or beta-blockers were not significantly different between the two groups. With regard to RA characteristics, the two groups differed only for greater use of hydroxychloroquine in the csDMARD group (Table 1).

**Table 1:** Baseline characteristics of the study population

Variables	csDMARDs (***n***=43)	TNFi (***n***=64)	***P***-value
**Cardiovascular disease risk factors**			
Age, median years (IQR)	58.6 (53.0, 66.0)	58.1 (49.3, 67.0)	0.839
Female sex	33 (76.7)	54 (84.4)	0.321
Obesity	5 (11.6)	7 (10.9)	0.999
Hypertension	19 (44.2)	30 (46.9)	0.784
Anti-hypertensive drug	17 (39.5)	28 (43.8)	0.784
Smoking status, ever	18 (42.9)	30 (46.9)	0.684
Dyslipidaemia	30 (40.2)	34 (59.8)	0.085
Current statin use	13 (34.2)	10 (15.9)	0.033
Diabetes mellitus	3 (7.0)	3 (4.7)	0.676
Anti-diabetic medication	1 (2.3)	1 (1.5)	0.999
CVD risk factors, median (IQR)	2 (1, 3)	2 (1, 3)	0.199
**Rheumatoid arthritis characteristics and treatment**			
RF and/or ACPA positive	28 (65.1)	33 (51.6)	0.165
Disease duration, median years (IQR)	14.1 (11.5)	15.4 (10.5)	0.538
Methotrexate	38 (88.4)	52 (81.3)	0.192
Leflunomide	5 (17.9)	12 (19.0)	0.999
Hydroxychloroquine	9 (31.0)	5 (7.8)	0.009
Prednisone > 5 mg daily	7 (7.7)	5 (5.5)	0.823
NSAIDs	6 (20.7)	22 (34.4)	0.227

*ACPA*, anti-citrullinated peptides antibodies; *csDMARDs*, conventional synthetic disease-modifying anti-rheumatic drugs; *IQR*, interquartile range; *NSAIDs*, non-steroidal anti-inflammatory drugs; *RF*, rheumatoid factor; *TNFi*, tumour necrosis factor inhibitors. All data reported as absolute numbers (percentage) otherwise specified. P-value refers to the chi-squared or Fisher test for categorical variables or independent samples T-test for continuous variables

### Reduced aortic stiffness progression with TNFi compared to csDMARDs

The two groups did not differ significantly for baseline AoSI (5.95±3.73% vs 6.08±4.20%, *p*=0.867). However, follow-up AoSI was significantly increased in the csDMARDs group (mean difference 1.00%, 95% CI 0.59, 1.42; *p*<0.0001) but not in the TNFi group (mean difference 0.15%, 95% CI −0.28, 0.60, *p*=0.477). Patients on TNFi had significantly lower follow-up AoSI than the csDMARD group (6.11±0.18% vs 7.13±0.22%; adjusted mean difference, aMD −1.02, 95% CI −1.581, −0.457, *p*<0.001; ANCOVA).

### Interaction of treatment and CVD risk factors on aortic stiffness

**Fig. 1:**
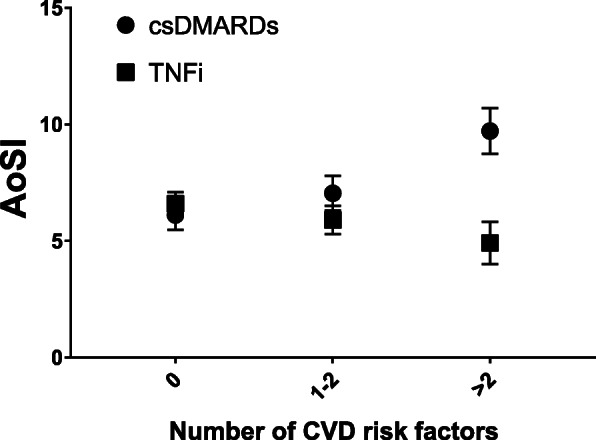
Interaction between treatment and cardiovascular disease risk factors on aortic stiffness index (two-way ANCOVA). Data are presented as adjusted estimated means of follow-up AoSI (bullets and squares) and their 95% confidence intervals (vertical error bars). csDMARDs, conventional synthetic disease-modifying anti-rheumatic drugs; TNFi, tumour necrosis factor inhibitors

**Fig. 2:**
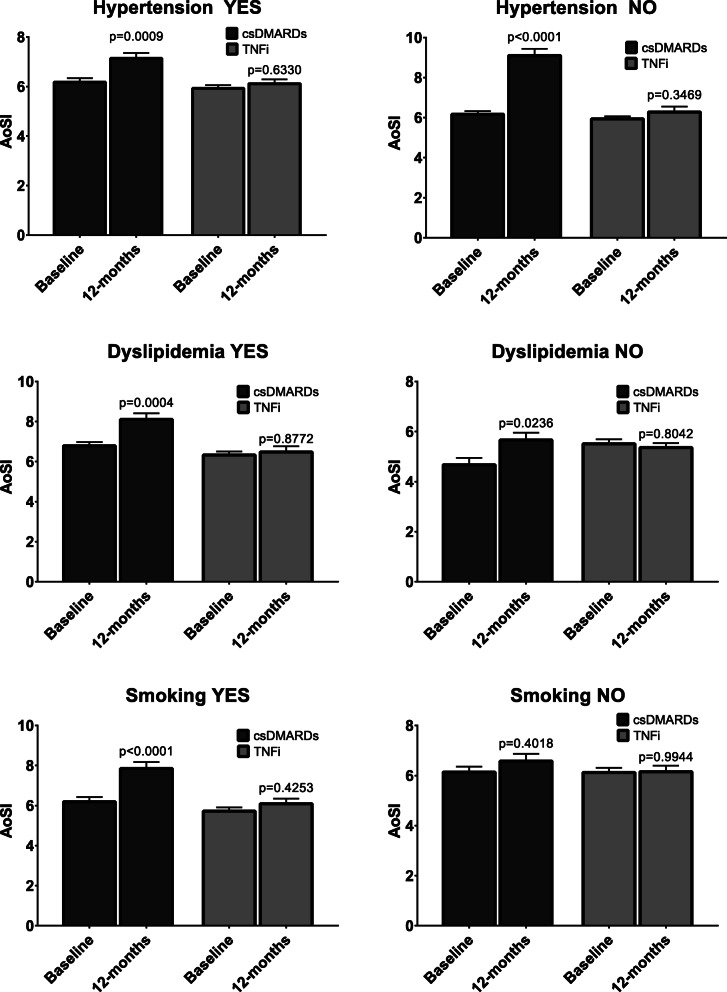
Grouped bar charts representing means (bars) and standard errors of the mean (vertical error bars) of follow-up aortic stiffness index (AoSI) values according to treatment group and cardiovascular disease risk factors. csDMARDs, conventional synthetic disease-modifying anti-rheumatic drugs; TNFi, tumour necrosis factor inhibitors. *P*-value refers to paired samples T-test

There was a statistically significant two-way interaction between the treatment group and the number of CVD risk factors on AoSI at follow-up, while controlling for baseline AoSI, age and SBP (*p*<0.0001, η^2^=0.038). Follow-up AoSI was lower in the TNFi compared to csDMARDs group (Fig. 1) both when CVD risk factors were 1–2 (aMD −1.143, 95% CI −2.102, −0.185, *p*=0.019) and when CVD risk factors were more than two (aMD −4.806, 95% CI −6.128, −3.484, *p*<0.001). We also compared the effect on aortic stiffness of TNFi and csDMARDs therapy across RA patients according to the presence of the most prevalent CVD risk factors in our study population: hypertension, dyslipidaemia and smoking. Adjusted AoSI means at follow-up were significantly higher than baseline in the csDMARDs group but not in the TNFI group (Fig. 2).

### Changes in lipids, glucose and blood pressure induced by DMARD therapy.

Overall, favourable changes in lipids and glucose after DMARD therapy were found (Table 2). There was a significant reduction in TC, VLDL, LDL and HDL in the csDMARDs group. SFG was reduced both in the csDMARDs and TNFi group. However, blood pressure (both SBP and DBP) was significantly increased in the csDMARDs group, whereas DBP was significantly decreased in the TNFi group. We found no significant correlations between changes in AoSI and serum lipids, glucose or arterial blood pressure.

**Table 2:** Longitudinal changes in CVD risk factors and RA disease activity according to treatment group

	csDMARDs			TNFi		
	Baseline	12 months	***P***-value	Baseline	12 months	***P***-value
**Cardiovascular disease risk factors**						
TGL	123 (68)	114 (50)	<0.001	113 (48)	106 (46)	0.023
LDL	122 (32)	112 (19)	<0.001	125 (26)	126 (30)	0.535
HDL	69 (12)	66 (10)	<0.001	74 (22)	74 (19)	0.609
TC	212 (3)	200 (19)	<0.001	221 (30)	218 (38)	0.161
SFG	88 (8)	85 (15)	<0.001	94 (13)	91 (11)	0.001
SBP	130 (19)	135 (19)	<0.001	136 (19)	135 (21)	0.152
DBP	80 (10)	83 (11)	<0.001	86 (10)	82 (10)	<0.001
BMI	25.5 (4.5)	25.5 (4.5)	0.442	25.6 (4.1)	25.6 (4.1)	0.226
**Rheumatoid arthritis disease activity**						
ESR	20 (20)	16 (17)	<0.001	20 (17)	19 (14)	0.001
CRP	5.7 (8.2)	4.6 (8.7)	<0.001	2.40 (2.51)	2.49 (2.15)	0.410
CDAI	10 (10)	10 (10)	0.173	9 (7)	11 (10)	<0.001
DAS28	2.05 (1.35)	2.54 (1.36)	0.002	2.64 (0.77)	2.81 (1.23)	0.108

*AoSI*, aortic stiffness index; *BMI*, body mass index, *CDAI*, clinical disease activity score index; *CRP*, C-reactive protein; *csDMARDs*, conventional synthetic disease-modifying anti-rheumatic drugs; *DAS28*, disease activity score-28; *DBP*, diastolic blood pressure; *ESR*, erythrocyte sedimentation rate; *HDL*; high-density lipoprotein; *LDL*, low-density lipoprotein; *SBP*, systolic blood pressure; *SFG*, serum fasting glucose; *TC*, total cholesterol; *TGL*, triglycerides; *TNFi*, tumour necrosis factor inhibitors. All data reported as mean (standard deviation). P-value refers to paired samples t-test comparisons between values at baseline and 12 months

## Discussion

The original finding of this study is showing that arterial stiffness progression can be hampered by TNFi not only in early but even in long-standing RA. Those individuals show a greater number of CVD risk factors than early RA patients [29] and higher CVD mortality than the general population [1]. Hence, control of CVD risk in such patients is the most important outcome to achieve along with control of disease activity.

Prior studies on the effect of TNFi on arterial stiffness focused on early RA patients with short disease duration and relatively low CVD risk. We noticed favourable effects in terms of reduction of aortic stiffness with TNFi compared to csDMARDs in a cohort of patients with several CVD risk factors. TNFi can reduce endothelial dysfunction and reduce carotid intima-media thickness [30]. Skin microvascular responses assessed by laser Doppler imaging improved in patients with active RA and no previous history of CVD who responded to TNFi or MTX [31]. Short-term treatment with TNFi also increased circulating endothelial progenitor cells concurrently with a proportional decrease of disease activity [32]. Our results, along with the previous evidence, are consistent with the hypothesis that the vascular-protective effect could be effectively achieved by inhibition of TNF [33].

The effect of TNFi on arterial stiffness in RA was deemed to be independent of the reduction of systemic inflammation in patients with very high disease activity [19]. Herein, the proportion of patients with high disease activity was very low; hence, we provided further evidence that the beneficial effect of TNF-alpha inhibition on arterial stiffness goes beyond the DMARD-associated reduction of systemic inflammation. Indeed, we also found no association between inflammatory markers or disease activity scores and aortic stiffness. However, the relationship between disease activity and AoSI was difficult to ascertain as all changes in disease activity scores were subtle and non-clinically meaningful, as they did not lead to treatment changes.

The third result of our study is that TNFi may be more beneficial than csDMARDs in presence of some CVD risk factors such as hypertension, dyslipidaemia and smoking. Although traditional CV risk factors alone do not explain the heightened risk of CVD in RA [34], a meta-analysis confirmed hypertension, type 2 DM, smoking and hypercholesterolaemia as key traditional factors increasing the risk of CVD in RA [35]. Hence, we analysed changes in aortic stiffness according to the presence of each of these CVD risk factors, except for DM due to the scarcity of patients with DM in our study population.

There was a sharp cardiovascular benefit of TNFi over csDMARDs in hypertensive RA patients. Moreover, TNFi therapy significantly decreased DBP values at follow-up, while SBP and DBP were both increased in the csDMARDs group. Essential hypertension was reported in up to 57% of patients with RA and can predict CV events (HR 3.67, 95% CI 2.0, 6.4, *p* = 0.001) [36]. Several small studies support the potential BP-lowering effect of TNFi in RA patients [37]. Nonetheless, in a US epidemiological study of RA patients, treatment with TNFi did not reduce the risk of incident hypertension compared with non-bDMARDs [38]. Interestingly, we showed that TNFi decreased AoSI and DBP also in normotensive RA patients, suggesting that the main driver of decreased BP is the TNFi-mediated favourable effect on arterial stiffness.

Patients with RA and dyslipidaemia on TNFi also showed reduced arterial stiffness. Moreover, 1 year of therapy with TNFi did not increase blood lipids, a finding that is in line with a meta-analysis of 25 RCTs of patients with chronic inflammatory arthritis that failed to demonstrate an effect of TNFi on TC, HDL-C and LDL-C [39]. Similar results were obtained by a recent RCT investigating the cardiovascular safety of tocilizumab against etanercept [40]. Conversely, there was a significant reduction of lipids with csDMARDs despite worse results on the progression of aortic stiffness, suggesting that arterial stiffness in RA may be scarcely associated with serum lipid levels. This finding can be partially explained by the higher number of patients taking HCQ in the csDMARD group. Although HCQ confers limited efficacy on disease activity and progression of RA, HCQ increases HDL and reduces levels of TC, LDL-C and triglycerides [41]. Additionally, we noticed decreased glucose across treatment groups, consistent with the lower incidence of diabetes with the use of HCQ [41, 42] or TNFi [42] amongst RA patients.

Finally, we showed an effect on arterial stiffness of TNFi therapy in smokers. Cigarette smoking is the strongest known lifestyle or environmental risk factor for RA [25, 43–45] and RA treatment failure [46]. Moreover, smoking can damage the vascular wall, possibly leading to impaired prostacyclin production and enhanced platelet-vessel wall interactions [47]. This can reduce the elastic properties of the aorta, resulting in stiffening and trauma to the wall [48]. Smoking, as well as passive exposure to smoke, impairs endothelium-dependent vasodilation of normal coronary arteries and reduces coronary flow reserve [49–53]. Smoking can also potentiate the endothelial dysfunction induced by hypercholesterolaemia [54].

### Study limitations and strengths

The main strength of this study consists of including a real-life cohort of RA patients with long-standing disease, several CVD risk factors and stable treatment. This kind of patient represents most patients we manage daily in our outpatient clinics. We used a prospective design, stringent entry criteria and a reliable method for the assessment of aortic stiffness which could be easily implemented in clinical practice. With regard to study limitations, we have to underline the relatively small sample size and the cross-sectional design of the study (patients were not randomized for treatment arms). Disease activity and lifestyle modifications are difficult to evaluate outside a clinical trial, but the vast majority of patients had stable disease activity and behavioural changes were very rare and of minimal clinical impact. Furthermore, we certainly cannot draw conclusions on RA patients on non-TNFi biologics as they were not included. Moreover, we could not substantiate a reduction of CVD events in RA patients with decreased arterial stiffness as the study was not powered for this outcome. Finally, smoking status was recorded as a binomial variable (ever vs never) and the number of pack-years was not calculated.

## Clinical implications and conclusions

Long-standing RA is commonly managed in rheumatology outpatient clinics. Our results seem to indicate that TNFi treatment could be associated with reduced arterial stiffness in patients with established, long-standing RA with several CVD risk factors. Although long-term TNFi therapy can be challenging due to the high CVD burden, our data encourage the assessment of AoSI in RA patients and maintain TNFi therapy whereas AoSI is abnormally high. This can be particularly relevant in such RA patients at high CVD risk.

## Data Availability

Data sharing is not applicable to this article; please contact the corresponding author for data requests.
